# Optimizing winter sportswear design and service priorities in China: a multi-model assessment

**DOI:** 10.3389/fspor.2025.1627600

**Published:** 2025-10-21

**Authors:** Kun Yang, Wenduo Liu, Zhengxue Song

**Affiliations:** ^1^ Liaoning Institute of Basic Medical Sciences, Shenyang, China; ^2^ Department of Sports Science, College of Natural Science, Jeonbuk National University, Jeonju, Republic of Korea; ^3^ College of Sports Science, Shenyang Normal University, Shenyang, China

**Keywords:** winter sportswear, requirement attributes, priority, IPA, borich, LF

## Abstract

**Background:**

People who maintain regular outdoor exercise in winter face many environmental and climatic challenges. Therefore, it is crucial to clearly prioritize the attributes of consumer demand for winter sportswear.

**Purpose:**

This study aims to identify the ranking of attributes that Chinese consumers of outdoor sports products value in their demand for winter sportswear.

**Participants and methods:**

This study collected data from sports enthusiasts in China's cold winter regions through an online questionnaire (the final effective data set contains 483). The scale collected attribute data on consumer needs for sportswear functions, pricing, service, design, and brand. Each survey includes consumer evaluations of the importance and performance of demand attributes. Demographic surveys include information on age, purchasing experience, and recent requirements. Finally, the results are statistically analyzed using *t*-test, IPA, Borich needs, and LF.

**Results:**

Top Priority includes color, ergonomics, logo and accuracy (shown as priority in IPA, Need and FL results). Second Priority includes logistics, packaging, return & exchange policy (shown as priority in Need and FL results). Third Priority includes technical cost, fabrics cost and quality control cost (only shown as priority in IPA result).

**Conclusion:**

The top three attributes that consumers care about most in the Chinese winter sportswear market are, in order of priority: design, service and pricing.

## 1 Introduction

The winter climate poses significant challenges to outdoor sports enthusiasts in maintaining their exercise habits. Advances in sportswear technology have further promoted the diversification and refinement of consumer demand for winter product functions (1). At the same time, the global sportswear market is slowing down (2), and the effective use of corporate resources has become the key to competition. Since the production cost of winter sportswear is generally higher than that of spring and summer models (such as high-insulation materials and complex processes) (3, 4), extensive operations will magnify the risk of resource waste. Decision-making theories indicate that consumer decision-making is a continuous process (5), while understanding and satisfying consumer needs is widely regarded as a key factor for products or services to achieve market success (6). If companies are unable to accurately position demand, it will lead to rising production costs, inventory backlogs, and ultimately a loss of market share under pressure from high-performing competitors (7). To resolve this conflict, companies should achieve precise matching through a collaborative mechanism for demand analysis and resource allocation (8). The Kano model proposes that analyzing consumer needs should follow three sequential steps: identifying attributes, determining priorities, and classifying attributes (9, 10). The attributes here are defined as tangible or intangible characteristics of a product or service perceived by consumers (11), directly reflecting their genuine needs. Means-end chain theory also emphasizes that attributes can lead to positive or negative consequences in consumer decision-making (12). By precisely targeting sets of positive attributes, companies can proactively avoid disconnects between business operations and consumer needs, reduce wasteful resource allocation, and build sustainable competitive advantages and market success.

Based on the theoretical framework, this study focuses on the needs of Chinese winter sportswear consumers and defines attributes as tangible and intangible characteristics that Chinese winter sportswear consumers can directly perceive, including function, pricing, design, service, and brand (13–17). For instance, to build sustainable competitive advantages in the fiercely competitive sportswear market, some premium athletic apparel companies are developing a synergistic, multi-dimensional strategy: collaborating with renowned designers to enhance the aesthetic appeal of their brand logos; maintaining market exclusivity through high-tech functionality and strategic pricing; and complementing these efforts with luxury services to precisely respond to and lead increasingly diverse consumer demands (17). By conducting a structured analysis of the demand attributes and employing a composite evaluation model to achieve precise prioritization (18), we extract high-priority attribute groups as the core demands driving the market.

The four prioritization models commonly used in business research currently include the importance-performance analysis model (IPA), the Locus for Focus model (LF), the Borich needs assessment model, and the independent sample *t*-test model (*t*-test) (19). It should be noted that each model has specific application scenarios and methodological limitations. The *t*-test can only verify the statistical significance of differences in importance (*I* value) and expressiveness (*P* value), but cannot directly show the priority ranking of attributes. IPA divides the area into “Sustain Resources”, “Increase Resources” and “No change in Resources”, “Curtail Resources” areas through a two-dimensional matrix, which can be used to initially locate the required attributes (20). LF uses an incremental analysis method and is particularly good at identifying key improvement areas of “High importance—High discrepancy”, and can generate improvement recommendations with a level of 1–4 (21). LF is superior to IPA in terms of improvement direction assessment, but lacks IPA's regional positioning function (22). It is worth noting that the above three models cannot quantify the contribution value of demand attributes. However, the Borich demand assessment model can accurately measure the contribution of each attribute through importance-expressiveness weighting (23). Therefore, it is recommended to integrate the *t*-test, IPA, LF, and Borich Needs model to balance the natural limitations of each model through Redundancy analysis (24).

There is currently a significant seasonal research gap in the study of sportswear consumption (25). Existing research has focused on general functional attributes, often investigating conflicting seasonal needs such as perspiration resistance and wind resistance together, lacking a precise analysis of the matching of sports scenarios and consumer needs in different seasons (26). Researchers generally fail to recognize the importance of seasonal pricing strategies, ignoring the difference in the cost of clothing between winter and spring and summer and the impact of winter service elements on consumer demand (27, 28). The lack of these studies weakens the practical value of existing studies in guiding companies in making precise strategic decisions.

This study aims to identify the attributes of consumer demand for winter sportswear and make up for the lack of the existing seasonal dimension. Furthermore, it uses the composite model redundancy analysis method to accurately screen the decisive factors for Chinese consumers when purchasing winter sportswear. It provides a scientific basis for enterprises to achieve intensive production, improve marketing efficiency and optimize consumer decision-making.

## 2 Materials and methods

### 2.1 Research design and process

**Figure 1:**
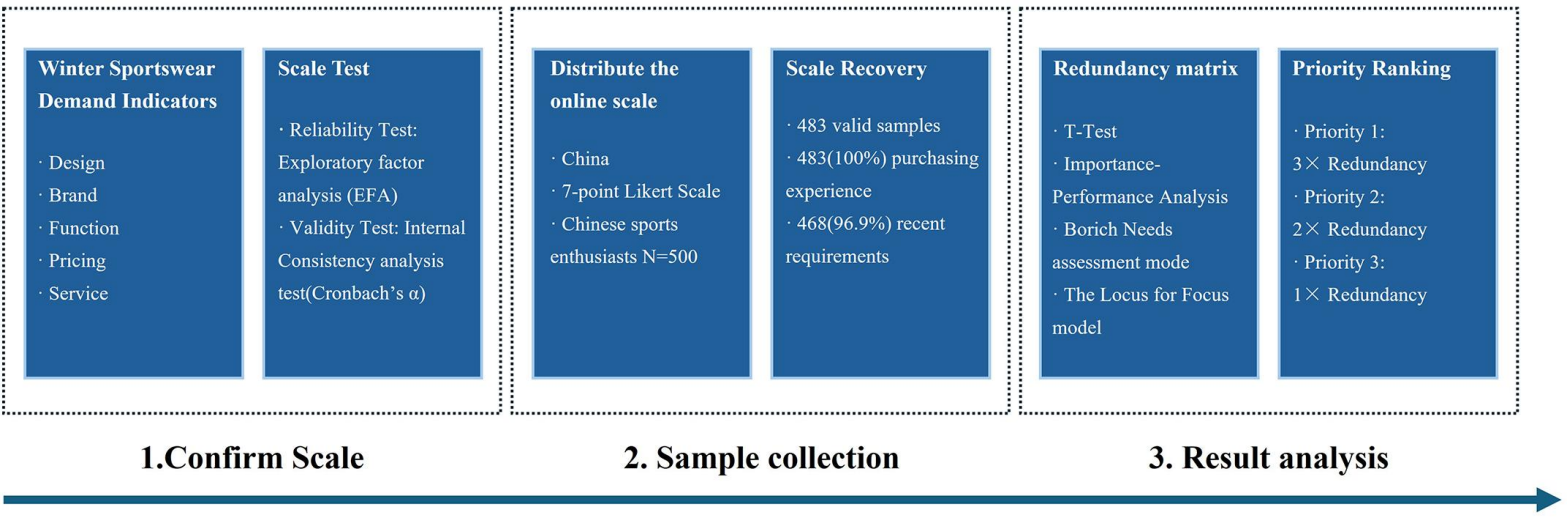
Design and process.

The design and process of this study are shown in Figure 1. This study employs purposive sampling, primarily targeting sports enthusiasts in cold winter regions such as Northeast China, North China, and Northwest China. All respondents must be consumers aged 16 or older who have purchased winter sports apparel within the past year. First, based on previous research, core indicators of consumer demand for winter sportswear were selected, and a scale was designed to pre-test 100 target consumers. Exploratory factor analysis (EFA) was used to optimize the item structure and reliability and validity. Subsequently, the researchers distributed the official online scale to 500 Chinese sports enthusiasts and standardized the subjects’ understanding and operational procedures through explanatory materials before filling in the scale to reduce data bias. After the scale was returned, the researchers eliminated invalid samples with duplicate options or missing information, and verified the stability of the data through formal EFA testing to confirm a valid dataset of 483.

On this basis, the study then proceeds to a multi-dimensional analysis: demographic statistics (describing the attributes of the sample base), *t*-test matrix (comparing differences in consumer demand), IPA matrix diagram (locating the improved attributes in the second quadrant; The intersection of the abscissa *P* and ordinate *I* is determined using the average of the respective axes) (20), LF matrix diagram (locates the improvement attributes in the first quadrant; The intersection of the abscissa *I-P* and ordinate *I* is determined using the average of the respective axes) (21), and Borich demand value (measures the priority of consumer demand; Borich requirement degree $=\Sigma \left(\frac{I-P}{N}\right)\times \bar{I}$) (23).

Ultimately, the priority-redundancy matrix clarifies the resource allocation strategy and achieves the research objective of demand mining to priority ranking and decision support.

### 2.2 Research tools and testing

The measurement tool used in this study is based on Table 1, with modifications and additions. The scale uses a Likert-7 point scale, with 7 indicating strong agreement and 1 indicating strong disagreement. During pre-testing, the potential relationship between the scale variables and the data structure was tested using EFA. The default value is to delete items with a factor load <0.6. One item was deleted from the pre-survey (*n* = 100). After the formal scale was recovered, the reliability and validity of the scale were confirmed using Cronbach's Alpha, principal component analysis (PCA), and EFA. Based on the valid data (*n* = 483), the scale is divided into five first-level indicators: functions, pricing, service, design, and brands. The 25 secondary indicators are as follows: Functions (7 indicators, nos. 1–7), Pricing (3 indicators, nos. 8–10), Service (4 indicators, nos. 11–14), Design (4 indicators, nos. 15–18), Brands (7 indicators, nos. 19–25). The results are shown in Table 1.

**Table 1:** Source of core indicators.

Attribute		Reference
Functions	1. Lightweight	Gorade et al., 2021; (3) Luo et al., 2021; (4) Skomra, 2006; (42) De Raeve and Vasile, 2016; (43) Kim et al., 2018; (13) Hayes and Venkatraman, 2016; (44)
	2. Fabric Stretch	
	3. Cold Resistance	
	4. Comfort Sensation	
	5. Cross Scenario	
	6. Windproof & Waterproof	
	7. Easy Care	
Pricing	8. Technical cost	Yan et al., 2008; (45) Choi, 2017; (14) Kapelko and Oude Lansink, 2014; (46)
	9. Fabrics cost	
	10. Quality Control cost	
Service	11. Membership Privileges	Kim and Lennon, 2005; (47) Hinkka et al., 2015; (15) Wood, 2001; (48) Wallenburg et al., 2021; (49)
	12. Logistics	
	13. Return & Exchange Policy	
	14. Packaging	
Design	15. Color	Goldschmied et al., 2023; (50) Salmon and Macquet, 2019; (51) Aakko and Niinimäki, 2022; (16) Walsh et al., 2019; (52)
	16. Ergonomics	
	17. Logo	
	18. Accuracy	
Brands	19. High Quality image	Lu and Xu, 2015; (53) Swimberghe et al., 2014; (54) Lim et al., 2016; (17) Tong and Hawley, 2009; (55)
	20. Trust	
	21. Awareness	
	22. User imagery congruity	
	23. Dependence	
	24. Exposure	
	25. Exclusive Products	

Reliability is measured using Cronbach's alpha. The validity test uses EFA to measure the factors and factor loadings contained in each attribute. In the EFA test, this study uses a factor load ≥0.6 as a reference value, which is higher than the international standard. Items with factor loads below the standard are deleted, and the next exploratory factor analysis is performed again until all factors reach the reference value. After three rounds of exploration factor analysis, all 7 items of brand equity were retained; 2 of the 9 items of functionality were deleted, leaving 7 items; all 4 items of design were retained; all 4 items of after-sales service were retained; and 1 of the 4 items of price was deleted, leaving 3 items.

**Figure 2:**
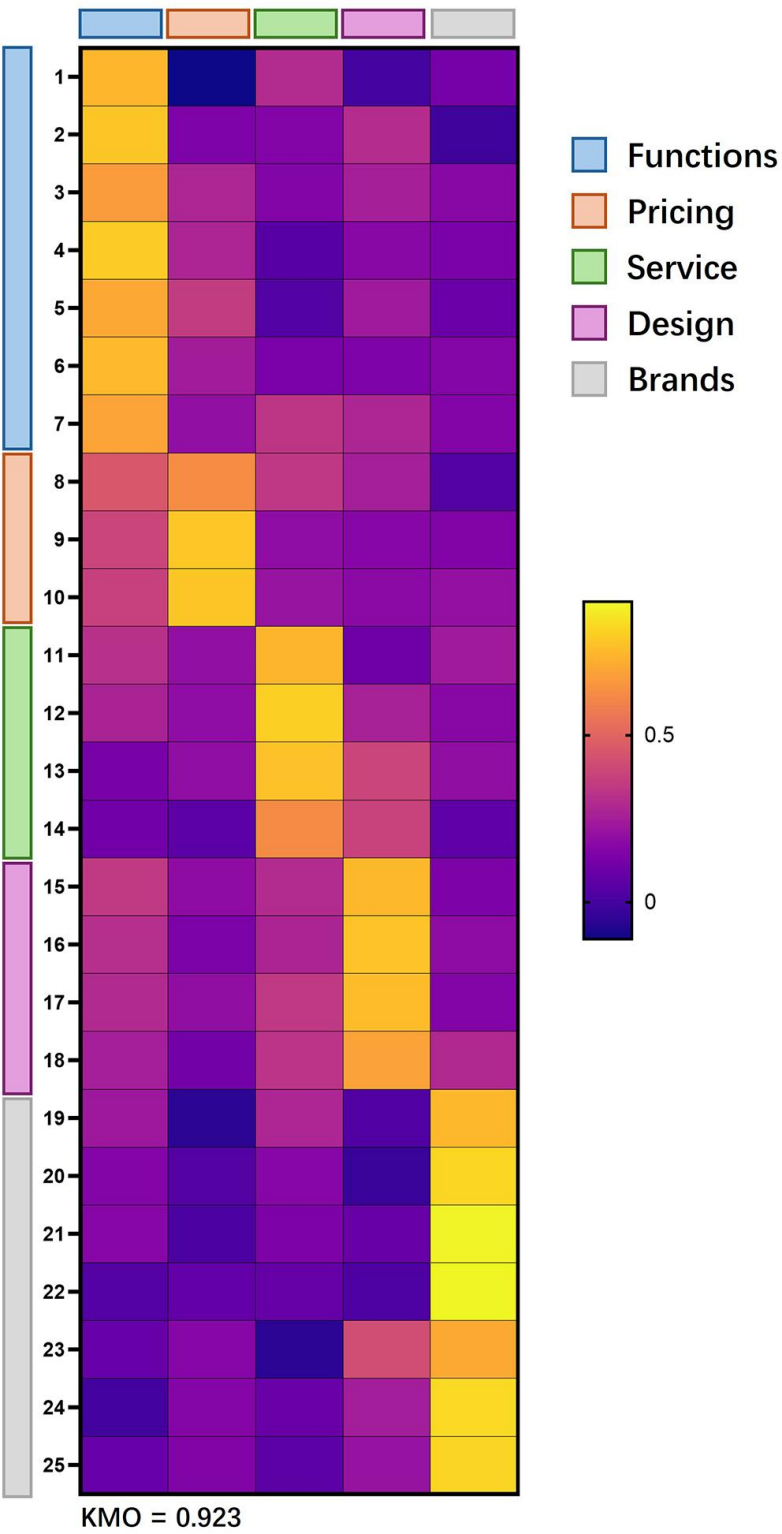
Results of reliability and validity test.

In the end, the sportswear selection attributes were reduced from 28 items to 25 items. The reliability test found that the Cronbach's α values of all secondary variables ranged from .879 to .928, all of which were greater than 0.7. The KMO value was 0.917, which met the preconditions for exploration factor analysis. The validity test found that the factor loadings within the five secondary variables were all greater than the standard value of 0.6. The above results prove that the questionnaire has good reliability and validity. The specific parameters are shown in Table 2; Figure 2.

**Table 2:** Reliability and validity test.

Attribute	Item	Functions	Pricing	Service	Design	Brands	Cronbach's α
Functions	1	0.739	−0.111	0.295	−0.003	0.121	.909
	2	0.780	0.141	0.154	0.298	−0.015	
	3	0.668	0.276	0.151	0.253	0.168	
	4	0.791	0.274	0.040	0.168	0.133	
	5	0.700	0.353	0.030	0.235	0.092	
	6	0.746	0.243	0.133	0.142	0.159	
	7	0.691	0.196	0.334	0.277	0.157	
Pricing	8	0.450	0.626	0.338	0.250	0.035	.902
	9	0.385	0.785	0.187	0.164	0.150	
	10	0.371	0.779	0.212	0.178	0.199	
Service	11	0.316	0.198	0.737	0.100	0.235	.872
	12	0.267	0.190	0.801	0.260	0.170	
	13	0.124	0.191	0.768	0.385	0.194	
	14	0.106	0.053	0.619	0.377	0.060	
Design	15	0.345	0.183	0.297	0.745	0.137	.925
	16	0.307	0.136	0.270	0.772	0.184	
	17	0.290	0.194	0.341	0.754	0.158	
	18	0.251	0.108	0.326	0.685	0.288	
Brands	19	0.227	−0.061	0.276	0.028	0.743	.932
	20	0.154	0.032	0.163	−0.028	0.819	
	21	0.164	0.015	0.137	0.084	0.887	
	22	0.033	0.073	0.079	0.020	0.894	
	23	0.080	0.166	−0.061	0.415	0.705	
	24	−0.005	0.159	0.088	0.247	0.828	
	25	0.081	0.147	0.053	0.207	0.815	
Variance		4.913	2.321	3.143	3.296	5.124	
Cumulative		19.650%	28.933%	41.505%	54.690%	75.185%	
KMO		.923					

### 2.3 Participants and sample

A total of 483 valid scale data were collected in this study, and data collection was completed by participants using their mobile phones to scan QR codes. All participants met the following criteria: 1) they had the consumption experience of purchasing Winter sportswear; 2) they volunteered to participate in this study. Before entering the questionnaire system, participants first needed to sign an informed consent form online before they could start filling out the scale. In addition, this research proposal has been formally approved after the ethical review by the institutional review board.

### 2.4 Data analysis

After preprocessing the data collected, SPSS27 was used for statistics (IPA, LF, Borich needs values and priorities–redundancy matrix). Descriptive statistics were used for demographic variables. The significance of the importance and expressiveness of the demand attributes was tested using an independent sample *t*-test with a significance level of *p* < 0.05.

## 3 Results

### 3.1 Demographic characteristics

All subjects had experience buying sportswear, and 96.9% (468) of them said they had a recent need to buy. The specific demographic characteristics are shown in Table 3. The power analysis (G Power 3.1, Germany) showed that a minimum of 105 participants were required to detect a medium effect size (d = 0.5) with an alpha of 0.05 and a statistical power of 95%. Thus, the sample size of this study fully meets the statistical power requirements.

**Table 3:** General characteristics of the participants.

Variable (*N* = 483)	Content	*N* (%)
Gender	Male	203 (42%)
	Female	280 (58%)
Age	16–29	223 (46.2%)
	30–39	104 (21.5%)
	40–49	83 (17.2%)
	50–59	63 (13%)
	≥60	10 (2.1%)
Purchasing experience	Yes	483 (100%)
	No	0 (0%)
Recent requirements	Yes	468 (96.9%)
	No	15 (3.1%)

### 3.2 The importance and performance of winter sportswear

**Figure 3:**
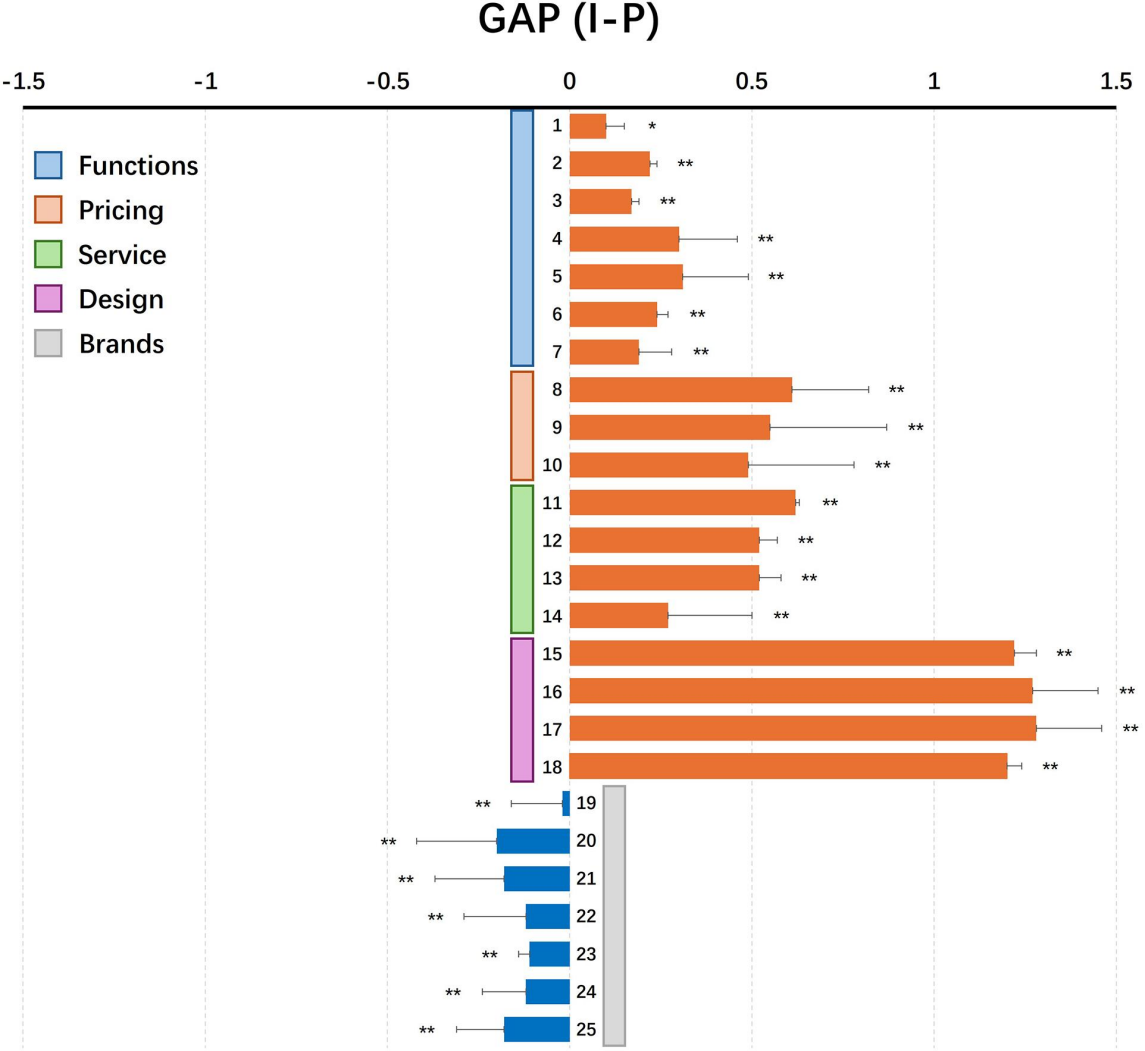
Results of the Gap level (importance and performance). Data was analyzed by *t*-test (*p < 0.05; **p < 0.01; ***p < 0.001).

This study used the *t*-test to evaluate the differences between importance and performance (Table 4; Figure 3). The results showed that there were differences between importance and performance for all 25 items (reference value *P* < 0.05). In addition, it was found that the GAP value for brands (19–25) was <0; the Gap values for function (1–7), pricing (8–10), service (11–14), and design (15–18) were >0.

**Table 4:** Results of importance and performance *T*-test.

Attribute	Item	Importance		Performance		Gap (I-P)		*p*
		M	SD	M	SD	M	SD	
Functions	1F	5.91	1.290	5.81	1.237	0.10	0.05	.025
	2F	6.16	1.068	5.94	1.084	0.22	0.02	.001
	3F	5.82	1.220	5.65	1.238	0.17	0.02	.001
	4F	6.04	1.058	5.75	1.219	0.30	0.16	.001
	5F	6.13	1.039	5.82	1.223	0.31	0.18	.001
	6F	5.9	1.166	5.66	1.200	0.24	0.03	.001
	7F	5.94	1.128	5.74	1.222	0.19	0.09	.001
Pricing	8P	5.79	1.182	5.5	1.391	0.61	0.21	.001
	9P	5.78	1.103	5.56	1.419	0.55	0.32	.001
	10P	5.72	1.193	5.57	1.479	0.49	0.29	.001
Service	11A	5.59	1.489	4.97	1.504	0.62	0.01	.001
	12A	5.69	1.325	5.07	1.375	0.52	0.05	.001
	13A	5.68	1.369	5.06	1.425	0.52	0.06	.001
	14A	5.92	1.478	5.21	1.250	0.27	0.23	.001
Design	15D	5.9	1.133	4.69	1.191	1.22	0.06	.001
	16D	5.85	1.096	4.59	1.280	1.27	0.18	.001
	17D	5.9	1.081	4.62	1.257	1.28	0.18	.001
	18D	5.8	1.207	4.61	1.245	1.20	0.04	.001
Brands	19B	5.34	1.513	5.36	1.369	−0.02	0.14	.001
	20B	5.06	1.555	5.27	1.338	−0.20	0.22	.001
	21B	5.06	1.628	5.24	1.439	−0.18	0.19	.001
	22B	4.92	1.616	5.04	1.445	−0.12	0.17	.001
	23B	5.3	1.441	5.41	1.409	−0.11	0.03	.001
	24B	5.13	1.484	5.26	1.362	−0.12	0.12	.001
	25B	5.3	1.450	5.48	1.321	−0.18	0.13	.001

### 3.3 Results of the IPA

**Figure 4:**
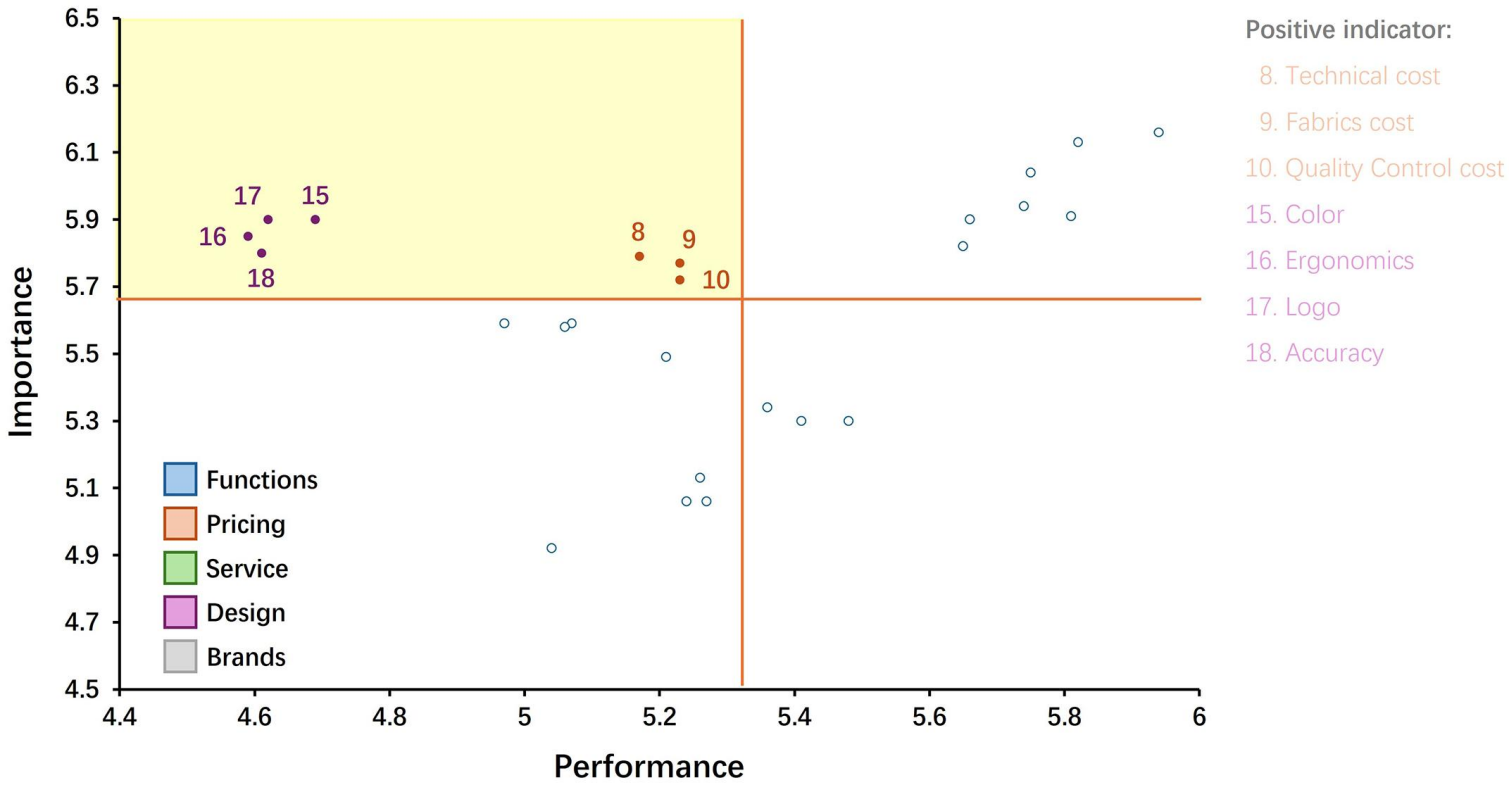
Results of the IPA.

The results of the IPA matrix are shown in Figure 4; Table 5. The second quadrant is the area of highest priority, where the price attributes (8–10) and design (15–18) appear. The seven attributes of price and design go into the priority-redundancy analysis.

**Table 5:** Four-Quadrant results of IPA.

Quadrant I Sustain resources	Quadrant II Increase resources	Quadrant III No change in resources	Quadrant IV Curtail resources
2	10	20	25
5	9	24	23
1	8	21	19
4	15	22	
7	17	14	
6	18	12	
3	16	13	
		11	

Quadrant I, High Importance + High Performance; Quadrant II, High Importance + Low Performance; Quadrant III, Low Importance + Low Performance; Quadrant IV, Low Importance + Low performance.

### 3.4 Results of the FL

**Figure 5:**
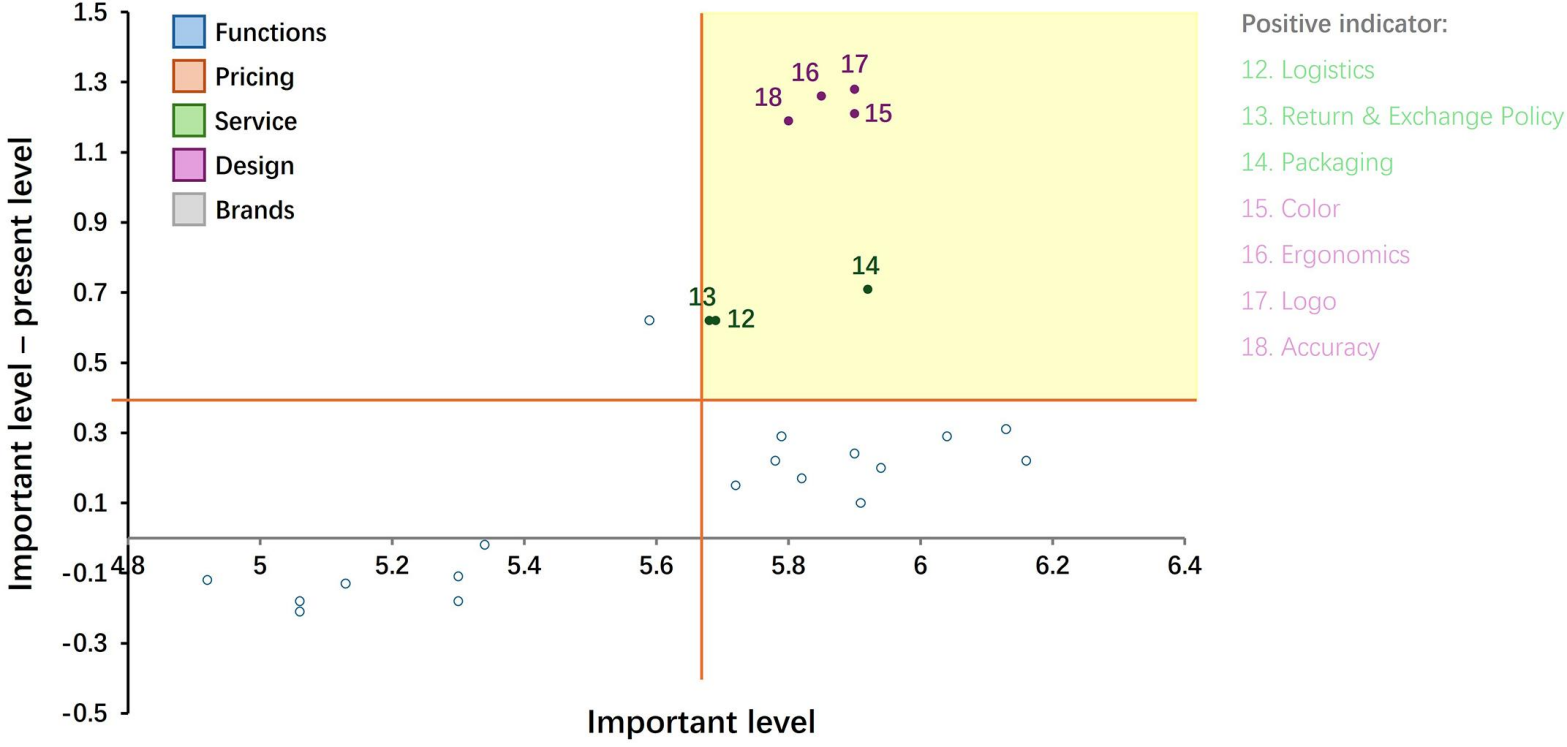
Results of the FL.

The results of the IPA matrix are shown in Figure 5; Table 6. The first quadrant is the area of highest priority, where services (12–14) and design (15–18) appear. The seven attributes of services and design enter into a priority-redundancy analysis.

**Table 6:** Four-Quadrant results of LF.

Quadrant I HH	Quadrant II HL	Quadrant III LL	Quadrant IV LH
17	11	19	5
16		23	8
15		24	4
18		22	6
14		20	2
13		25	7
12		21	9
		11	3
			10
			1

Quadrant I, High Importance + High Discrepancy; Quadrant II, Low Importance + High Discrepancy; Quadrant III, Low Importance + Low Discrepancy; Quadrant IV, High Importance + Low Discrepancy.

### 3.5 Results of the borich needs assessment

The results of the Borich needs assessment are shown in Table 7. The second quadrant of IPA and the first quadrant of FL are both high priority areas, with seven overlapping items. Therefore, the need analysis determines that the top seven values are of the highest priority. Design (15–18) and service (12–14) appear.

**Table 7:** Priority results of borich needs assessment mode.

The top seven list			The remaining list		
Order of priority	Need	Item	Order of priority	Need	Item
1	7.55	17	8	3.47	11
2	7.37	16	9	1.90	5
3	7.14	15	10	1.75	4
4	6.90	18	11	1.68	8
5	4.20	14	12	1.42	6
6	3.53	12	13	1.36	2
7	3.52	13	14	1.27	9
			15	1.19	7
			16	0.99	3
			17	0.86	10
			18	0.59	1
			19	−0.11	19
			20	−0.58	23
			21	−0.59	22
			22	−0.67	24
			23	−0.91	21
			24	−0.95	25
			25	−1.06	20

### 3.6 Results of the priority-redundancy analysis

The results of the Borich needs assessment are shown in Table 8. The seven highest priority items in the second quadrant of IPA 8, 9, 10, 15, 16, 17, 18 (Figure 4; Table 5); the seven highest priority items in the first quadrant of FL 12, 13, 14, 15, 16, 17, 18 (Figure 5; Table 6); and the seven items with the highest priority in the Need analysis 12, 13, 14, 15, 16, 17, 18 are included in the redundancy analysis (Table 7). It was found that the design items (15–18) were redundant three times, and the first priority improvement area was finally determined. The service items (12–14) were redundant twice, and the second priority improvement area was finally determined. The price (8–10) was redundant once, and the third priority improvement area was finally determined.

**Table 8:** Results of redundancy analysis.

Item	Need	Order of priority	IPA	Locus for focus	Suggest
8	1.68	Non	○	Non	Third priority IPA
9	1.27	Non	○	Non	
10	0.86	Non	○	Non	
12	3.53	6	Non	◇	Second priority need + FL
13	3.52	7	Non	◇	
14	4.20	5	Non	◇	
15	7.14	3	○	◇	Top priority IPA + FL + Need
16	7.37	2	○	◇	
17	7.55	1	○	◇	
18	6.90	4	○	◇	

## 4 Discussion

Research data shows that all respondents have purchased sportswear before, and 96.9% of them have a recent demand for winter sportswear. Analysis of the 25 items shows that there are significant differences in the dimensions of importance and performance. Specifically, in the dimension of design attributes, items 15, 16, 17, and 18 were identified as the top priority areas for improvement; items 12, 13, and 14 in the service attribute dimension were ranked as the second priority for improvement; and items 8, 9, and 10 in the price attribute dimension were ranked as the third priority for improvement (Table 8). This hierarchical result provides a clear direction for the optimal allocation of resources for winter sportswear companies.

Redundancy (×3) analysis: Based on comprehensive analysis results, color design, ergonomic design, brand logo design, and detail design should be ranked as the attributes with the highest priority for improvement (Table 8). The IPA analysis (Figure 4; Table 5) shows that these attributes have high importance and low performance characteristics. Consumers attach the greatest importance to unmet needs and urgently need improvement. At the same time, the LF analysis (Figure 5; Table 6) confirms that these needs are not only prominent in importance, but also highly controllable, which means that companies can achieve efficient improvement through reasonable resource allocation. The Need analysis (Table 7) further verifies the key position of these attributes. They rank among the top 4 out of 25 needs and are core attributes of consumer demand. These design attributes not only have a decisive impact on purchasing decisions, but they are also attributes over which companies have the best control. Prioritizing improvements in these areas will bring significant revenue growth. In the sportswear industry, these design elements are mainly concentrated in the production process, which directly affects the competitiveness of the product in the market. Therefore, it is recommended that companies focus on increasing investment in the design process, including measures such as introducing high-end design talent and increasing R&D budgets. In this way, companies can quickly respond to changes in market demand within controllable resources and accurately meet consumer expectations, thereby gaining the greatest competitive advantage.

The results of the research design attributes echo with the existing literature in multiple dimensions. In terms of color design, an empirical study by Roberts et al. revealed that the color design of sportswear not only significantly affects the choice preferences of male and female consumers, but also has a positive effect on competitive sports performance, which partially supports the results of this study (29). It is worth noting that the design requirements for winter sportswear are higher than those for ordinary clothing, as it needs to meet the dual needs of high-intensity sports scenarios and keeping warm. This characteristic is further explained in an ergonomic study, where consumers’ need for the ergonomic design of sportswear to improve sports comfort and performance is driving ergonomic design as a core manufacturing attribute of products (30). In addition, Oliveira et al. found that 79.6% of respondents found it difficult to move their bodies in cold environments, and that the ergonomics of clothing still needs further improvement, which partially supports the results of this study (31). In terms of brand logo design, the team logo creates a sustained driving force for consumption through emotional connections, and is demonstrating the value of consumption through the mass media (32). At the level of fine design, Li et al. quantified the positive impact of fine design on corporate cost control. Increasing design fineness can reduce operating costs and improve consumer satisfaction, which partially supports the results of this study (33). By cross-checking existing literature, this study extends the theory in three ways: First, it clarifies that color, ergonomics, brand logos, and detailed design constitute the core dimensions of consumer demand; second, it reveals a direct correlation between specific design solutions and the efficiency of enterprise resource allocation; and third, it matches the design attributes of sportswear with consumer usage scenarios through an analysis of winter scenes. These findings provide a decision-making basis for precise research and development in the sportswear industry that combines theoretical depth with practical value.

Redundancy (×2) analysis: According to the LF analysis, logistics, return policies and packaging should be given the highest priority for improvement. The Need analysis shows that these attributes rank 5th to 7th out of 25 indicators, indicating that they are important but not the most urgent. The IPA analysis further confirms that the current service performance is basically in line with consumer expectations and does not need to be improved for the time being. Redundant analysis confirms the significance of LF and Need results, while IPA results are meaningless. It can be determined that these service attributes, although they will affect consumers’ purchase decisions, have a relatively limited scope of influence. For service-sensitive consumers, companies should optimize these service links appropriately to remain competitive. For example, businesses that allow consumers to implement different interaction policies across physical and online channels, and logistics and distribution significantly increase the willingness to buy (34). While a lenient return policy meets consumer expectations, it also greatly increases operating costs. To control operating costs, companies should formulate differentiated return policies for clearance products and full-price products (35). In the context of homogeneous product quality, exquisite packaging can immediately stimulate consumer desire and increase willingness to pay. This effect has been verified in both the Chinese and American markets (36, 37) Given that winter sportswear sales highly overlap with the holiday season, companies need to accurately target gift consumers and adjust their marketing strategies in a timely manner. For non-service-oriented enterprises, it is recommended to set improvement priority to the second sequence. Accurately positioning consumer groups requires further in-depth measurement by the enterprise.

Redundancy (×1) analysis: Through a comprehensive analysis of process costs, fabric costs, and quality control costs, these attributes were found to be significant in the IPA analysis, but did not reach a significant level in the LF and demand analysis. As lower-level attributes of price, they mainly affect the pricing strategy of the enterprise. It should be emphasized that these cost elements are the key foundation for ensuring the quality of sportswear. Any reduction in related inputs to reduce prices will inevitably lead to a reduction in the profit margin or a decline in product quality, which in turn will quickly cause the enterprise to lose its competitive advantage in the market (38). Based on these attributes being in the third sequence of improvement priority, it is recommended that these price-related attributes avoid over-allocating the company's limited resources. Combined with the seasonal characteristics of the winter sportswear market, demand usually shows a rapid increase followed by a stable trend as the temperature changes (39, 40). Therefore, management strategies with low resource consumption, such as dynamic pricing mechanisms, on-demand production models, and accurate grasp of consumers’ psychological price thresholds, are recommended in order to achieve the optimal balance of maximizing corporate profits and price competitiveness (41).

In summary, the top three attributes that consumers care about most in the China winter sportswear market are, in order of priority: design, service and pricing. Comprehensive analysis shows that process cost, fabric cost and quality control cost are significant in IPA analysis, but not in LF and demand analysis. As lower-level attributes of price, these factors mainly affect the pricing strategy of enterprises. It should be noted that they form the key basis for quality assurance of sportswear. At the same time, the market demand for winter sportswear shows obvious seasonal characteristics, which will rapidly increase with temperature changes and then tend to stabilize. Therefore, it is recommended that companies adopt management strategies with low resource consumption, such as dynamic pricing mechanisms, on-demand production models, and accurate grasp of consumers’ psychological price thresholds, in order to achieve the optimal balance of profit maximization and price competitiveness. Given that these attributes are in the third sequence of improvement priorities, it is recommended that companies avoid over-allocating limited resources.

This study has certain geographical limitations. There is a significant temperature disparity between southern and northern China during winter, with southern regions even allowing outdoor exercise without the need for winter sportswear. The findings of this study are primarily based on survey data from colder regions of China (Northeast, North, Northwest, etc.). Due to geographical differences, the results may have limitations or lag in applying to areas like South China and Southwest China. Based on this, future research can be furthered in three directions: First, the market should be divided according to climate zones to conduct a more accurate analysis of demand prioritization; Second, more cross-scenario research is needed, especially an analysis of consumer demand prioritization when switching between indoor and outdoor sports in winter. Finally, it is recommended to expand to other seasons of sportswear demand prioritization research to establish a more comprehensive consumer demand prioritization system. In variable selection, the current model primarily focuses on core attributes such as design, service, and price, without fully incorporating emerging demand attributes like social recognition. Future research could expand the attribute scope to more comprehensively reflect market dynamics. Future studies may explore differences in demand structures across distinct consumer segments (e.g., professional sports, fitness, leisure) to enhance the explanatory power of winter sportswear attribute priorities.

## 5 Conclusions

In the China winter sports apparel market, the three attributes that consumers care about most are design, service, and pricing, in that order. Among these, design is an attribute of consumer needs that are unmet and urgently require improvement, and it is highly controllable. Companies can efficiently improve this attribute by adjusting design resource allocation, so it should be the primary focus for improvement. Although service attributes have a certain impact on purchasing decisions, their scope of influence is relatively limited. Companies can maintain their competitive advantage by targeting service-sensitive consumer groups with targeted optimizations. Service dimension ranks in the secondary improvement sequence. In contrast, improvements in pricing factors have the lowest priority, and companies are advised to avoid overemphasizing this area to ensure consistency between resource allocation efficiency and strategic priorities.

## Data Availability

The raw data supporting the conclusions of this article will be made available by the authors, without undue reservation.
